# Effectiveness of Kinesio taping and conventional physical therapy in the management of knee osteoarthritis: a randomized clinical trial

**DOI:** 10.1007/s11845-022-03247-9

**Published:** 2022-12-17

**Authors:** Shahul Hameed Pakkir Mohamed, Salem F. Alatawi

**Affiliations:** Department of Physical Therapy, Faculty of Applied Medical Sciences, University of Tabuk, Tabuk, 71491 Saudi Arabia

**Keywords:** Exercises, Kinesio taping, Knee osteoarthritis, TENS

## Abstract

**Background:**

Knee osteoarthritis (OA) is the most common kind of arthritis that occurs due to degeneration of the joint articular cartilage, producing pain, stiffness, and impaired movement. The objective of the study was to evaluate the short-term effectiveness of Kinesio taping (KT) plus conventional physical therapy (CPT) and CPT alone in subjects with knee OA.

**Materials and methods:**

Forty male subjects were divided into two groups at random using a parallel assignment, double-blinded study design, viz., KT with CPT (transcutaneous electrical nerve stimulation and exercise therapy), and CPT alone for the period of 6 weeks of treatment. At baseline, third, and sixth weeks, the following outcome measures were taken, such as pain intensity (NPRS), knee range of motion (goniometry), Western Ontario and McMaster Osteoarthritis Index (WOMAC), and the Time Up and Go (TUG) test.

**Statistical analysis:**

To reveal the patient’s demographic profile concerning the outcome parameters, a descriptive statistic was applied. Furthermore, two-way mixed ANOVA and Tukey HSD post hoc tests were used to analyze within and between-group comparisons in SPSS 20.0.

**Results:**

In both groups, pain and knee flexion were significantly improved during the 6-week period of interventions (*p* < 0.05). WOMAC and TUG test scores improved only in the KT plus CPT group.

**Conclusion:**

KT combined with CPT was found to be more effective than CPT alone in the third and sixth weeks of the treatment. In knee OA, this combination of treatments was found to reduce pain, enhance range of motion, and improve physical functioning.

## Introduction

Knee osteoarthritis (OA) joint disease most commonly occurs in the elderly population, and it causes significant pain and functional limitations [1, 2]. Reduced quadriceps muscular strength, decreased mobility, and loss of functional ability, all of which result in proprioception deficits, are among the existing predisposing factors [3, 4]. Manual therapy, physical therapy modalities, taping techniques, patient education and therapeutic exercises, orthosis, and, more recently, extracorporeal shock wave therapy (ESWT) are all used to treat knee OA [5, 6].

Kinesio taping (KT) is a therapeutic technique commonly used to treat knee OA [7]. KT is a high-stretch elastic adhesive material that allows the treated area to have free mobility [8]. Physical therapists (PTs) preferred the KT technique for knee OA rehabilitation due to its positive effects, such as increased quadriceps torque and pain management, as well as negative effects, such as decreased muscular performance and motor function [7, 9]. Because of its strong adherence, KT can be applied directly to the skin and left on for several days. The treatment is patient-friendly and reasonably easy to use in day-to-day life due to the low risk of skin irritation [10]. It was reported that using KT for 4 weeks reduced pain during the day, when people are more active, and resulted in a considerable reduction in the use of painkillers [11]. Moreover, KT stimulates a lymphatic drainage response, which aids the drainage of excess fluid in circulatory pathologies [12]. In line with this, KT application reduces excess heat by reducing friction, resulting in lifting the skin, and relieving pressure on the subcutaneous nociceptors, and it also acts as a good stabilizer [13]. Kinesio taping is a conservative therapeutic approach for the treatment of musculoskeletal disorders that has earned increased attention from physicians and physiotherapists in recent years. However, because of a scarcity of research studies, little is known about the impact on clinically relevant symptoms or the underlying physiological changes that may cause adverse consequences. As a result, the use and benefits of Kinesio taping are still hotly debated [14].

Exercise therapy (ET) is an important component of the treatment for osteoarthritis of the knee [15]. Through a mechano-transduction reaction, it affects articular cartilage metabolism and changes cartilaginous structure [16]. Several systematic reviews have revealed that all types of exercise can reduce knee OA joint discomfort and enhance physical function [15, 17]. Based on high-quality evidence of mid-term and long-term effects, ET is now suggested as the recommended treatment for individuals with knee OA [18]. A variety of transcutaneous electrical nerve stimulation (TENS) applications are used by PTs to manage pain in knee OA patients [19]. TENS can be used in combination with exercise or physical activity to help relieve pain, or it can be used as a separate pain treatment. Furthermore, TENS has been demonstrated to reduce pain associated with knee OA, perhaps leading to improved function, a higher quality of life, and the evasion of surgery [20]. We hypothesized that patients who received KT along with conventional physical therapy (CPT) over 6 weeks would have better ROM, improve functional status, and reduce pain compared with patients who received CPT alone in the treatment of knee OA. In addition, the findings of this study will aid in the development of evidence-based guidelines for using Kinesio tape with other therapeutic modalities to treat OA-related functional impairments. To date, no published study in Saudi Arabia has looked into the use of Kinesio taping in conjunction with conventional physical therapy in the treatment of knee osteoarthritis.

## Methodology

### Study design

The effectiveness of Kinesio taping and transcutaneous electrical nerve stimulation with exercises on knee osteoarthritis was evaluated using a parallel assignment; a double-blinded study design was used in this study.

### Protocol registration

The study was approved by the Ethics committee (IRB), University of Tabuk and is registered with ClinicalTrial.gov (NCT05151627).

### Subjects and Randomization

**Fig. 1:**
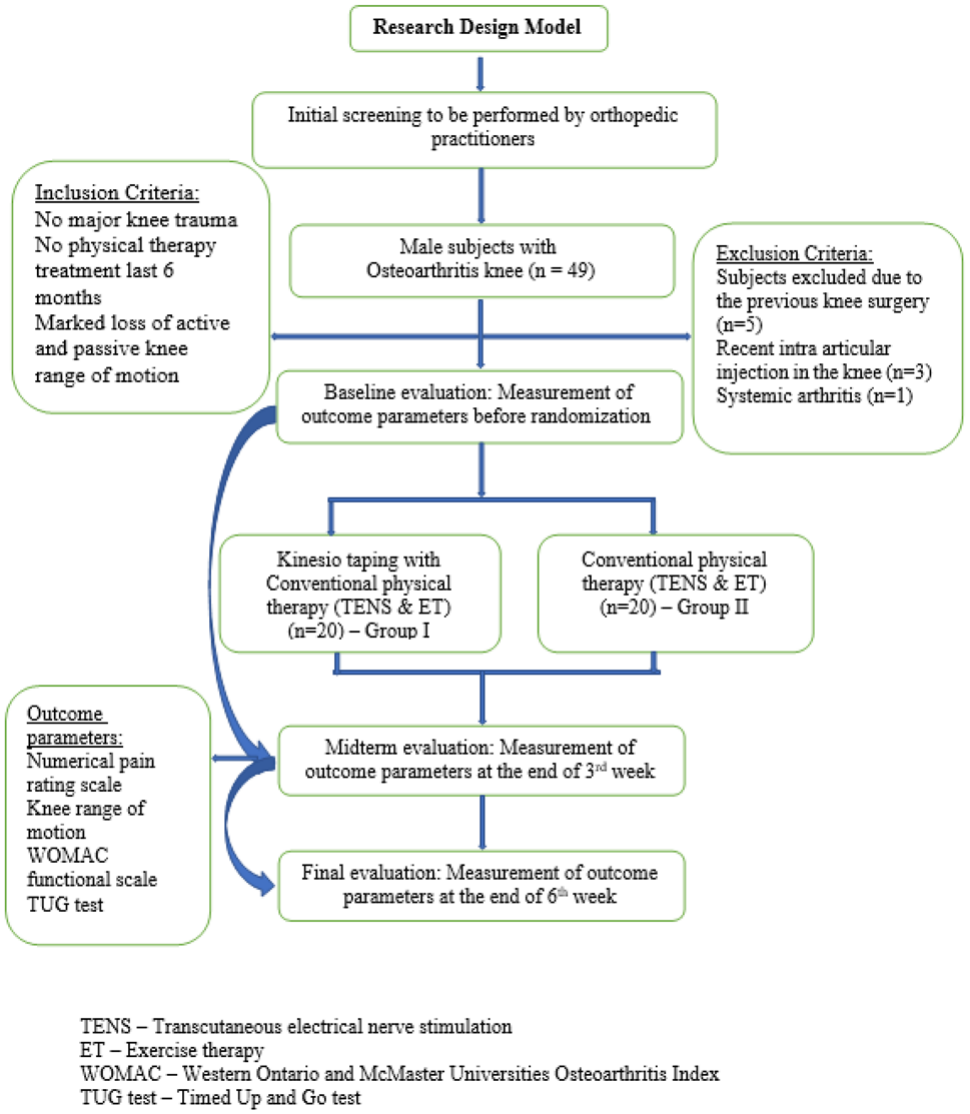
Flow chart diagram for the study design model

The study included all male subjects with knee pain who visited two government hospitals. All the subjects were referred to the physical therapy clinic by an orthopedic surgeon with a diagnosis of osteoarthritis (OA) in the knee. Both radiography and a clinical examination showed that they had OA changes in the knee joint. Among them, 49 subjects were between the ages of 40 and 70. Based on the application of both inclusion and exclusion criteria, nine patients were excluded, making a total of 40 subjects, and they participated in this study (Fig. 1). The total duration of this study was conducted for 7 months between July 2020 and January 2021. It is a randomized and double-blinded study.

### Inclusion and exclusion criteria

The participants in the study had to meet the following requirements: complaining of knee pain that has lasted longer than 3 months, and a pain level that is medium (pain score greater than or equal to 4) with no knee injuries, treatment not received in another physical therapy clinic in the past three months, painful range of motion (ROM), significant loss of active and passive ROM due to pain, stiffness, and swelling in the knee joint. Subjects were excluded if they had a history of knee surgery, any other neural, articular, or muscular conditions affecting lower limb function, a systemic arthritic condition, or had received an intra-articular injection or physical therapy in the past 3 months. Following a diagnosis of degenerative knee arthritis based on medical imaging such as X-rays, MRI, or bone scanning, patients are referred to the physical therapy department by an orthopedic consultant. An information leaflet explaining the study was given to the participants. Participants were enrolled and given the option to withdraw at any time by signing an informed consent form. During the initial evaluation, experienced PTs collected demographic information.

### Sample size calculation

The exercise effect on OA knee patients was investigated using the G power software, which detected a difference in WOMAC (Western Ontario and McMaster Universities Osteoarthritis) index stiffness from 3.66 ± 2.64 to 2.10 ± 2.26 [21]. Based on the impact size, each group needed twenty patients at 0.05 α level and 0.80 power value. Therefore, the total sample size of the sample was estimated to be 40 subjects (i.e., 20 in each group) in both groups. This total sample size would be increased to 49 for a potential dropout rate of 20%. Nine subjects were excluded due to knee surgery, recent intra-articular injection and systemic arthritis.

The formula used for sample size calculation is ${n}_{i}=2{\left(\frac{{Z}_{1-\alpha /2}+{Z}_{1-\beta }}{ES}\right)}^{2}$, with effect size (ES) = $\frac{\left|{\mu }_{1}-{\mu }_{2}\right|}{\sigma }$, where ${\mu }_{1}-{\mu }_{2}$ means the difference between two groups, $\sigma$ = SD, $\alpha$ is the selected level of significance, and $1-\beta$ is the selected power.

#### Outcome measurement tools

## Numerical pain rating scale (NRS)

The average pain intensity was measured during the past week, with zero representing no pain and ten representing the worst pain [22]. The NRS is an 11-point scale comprising a number from 0 through 10; 0 indicates “no pain,” and 10 indicates “worst imaginable pain.” Patients are instructed to choose a single number from the scale that best indicates their level of pain [23].

## Goniometric measurement for ROM

Both the knee flexion and extension ROM were measured while the subject was in a prone position. The measurement was done three times and the average result was obtained.

## Western Ontario and McMaster Universities Osteoarthritis Index (WOMAC)

The questionnaire was developed to measure treatment outcomes in patients with lower extremity osteoarthritis, such as pain, stiffness, and difficulty, and it employs a scale to categorize the difficulties in activities of daily living. For individuals with hip and knee osteoarthritis, the WOMAC scale is a valid and reliable outcome measurement [24].

## Timed Up and Go (TUG) test

The test combines static and dynamic balance to assess a person’s mobility. Getting out of a chair, walking three meters, turning around, and returning to the chair to sit. The international research OA society recommends a series of physical function measures (five performance-based) for individuals with hip or knee OA, including the TUG test [25]. The aforementioned outcome parameters are measured at three-time intervals: 0 week, 3 weeks, and 6 weeks.

## Interventions

All patients received 15 min of moist heat from a hydro collator pack wrapped in a soft towel put around the afflicted knee before beginning the main treatment. In one experimental group, KT followed by TENS was applied. After that, an exercise program was started. The other experimental group was followed by TENS and an exercise program. The purpose of this exercise program was to strengthen the muscles of the hip, knee, and ankle [26].

## Kinesio taping — 2 times per week for 6 weeks

**Fig. 2:**
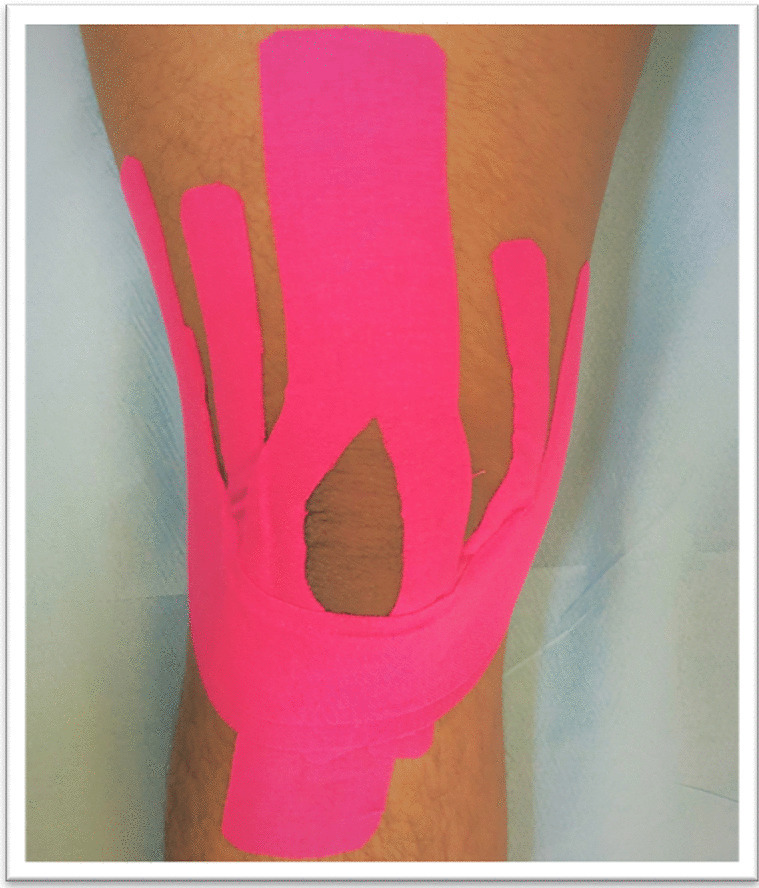
A view of the completed Kinesio taping (KT) application on the right knee

The professionally trained physical therapist applied Kinesio taping twice a week for the entire 6-week study period. The patient is in a supine position with full knee flexion. Before application, the skin surface was cleaned. This technique involved the use of two Y-cut strips and two I-cut strips. On the two Y-cut strips, the following effects and procedures were used: one to increase anterior thigh muscle function (“paper off” tension), and the other to lower the risk of knee joint effusion. The initial Y-cut strip of one end was placed on the rectus femoris muscle following the split ends passed through the medial and lateral sides of the patella and ended in the tibial tuberosity. The other Y-cut strip was placed just below the tibial tuberosity following the split ends, which passed through the medial and lateral sides of the patella and ended in the vastus medialis and lateralis muscles. The two Y-cut strips were tensioned at 0% for the first 5 cm, 10–15% for the middle area, and 0% for the last 2 cm. The adhesive is activated using the KT technique after each application. Two I-cut strips were applied one over the other on the patellar tendon toward the medial and lateral collateral ligaments to increase the mechanoreceptor stimulation, improve proprioception, and stabilize the knee. The subject was positioned in the supine position with the knee completely flexed. The first I-cut strip was applied directly to the inferior border of the patellar tendon with 100% tension following the adhesive tape was activated. Then the knee was extended to 20–30 degrees, with 75% of the tension applied until the tape reached the medial and lateral collateral ligaments, which was followed by adhesive activation. Finally, the subject was then instructed to extend the knee completely and direct the I-cut strip (about 10 cm) ends toward the posterolateral sides of the thigh with 0% tension and adhesive activation. Following that, the second strip was applied in the same method of application as the first, but it was applied lower and only covered half of the first (Fig. 2) [10, 27].

## Transcutaneous electrical nerve stimulation (TENS) — 2 times per week for 6 weeks

In our study, we used the following TENS device settings, such as the frequency of 32–50 Hz, the pulse width of 80 ms, and symmetrical biphasic waveform. The surface electrodes were placed on the medial and lateral superior borders of the patella, as well as the medial and lateral inferior borders of the patella. The electrodes were not placed on the quadriceps or the anterior leg muscles. The pairs of electrodes kept crossing to cover the large surface area. The device intensity was regulated by the attending physical therapist until subjects reported a bearable tingling sensation.

## Exercise therapy program

The exercise therapy training program continued for six weeks, two days every week. The recommended American Geriatrics Society exercise program was used in this study [28] with added exercises. The total duration of each exercise session was about 50 min, including warm-up-10 min, resistance exercise-20 min, balance and stability exercise-10 min, lower-limb stretching exercise-5 min, and cool-down exercise-5 min. The same assigned physical therapist supervised all of the sessions (Appendix).

### Statistical methods

To reveal the patients’ demographic profile concerning the outcome parameters, a descriptive statistic was applied. Furthermore, two-way mixed ANOVA and Tukey HSD post hoc tests were used to analyze within-group and between-group comparisons in SPSS 20.0.

## Results

Demographic variables were subjected to descriptive statistics in this study, and the findings are shown in Table 1. Patients in the KT plus CPT group were on average 61 years old, while those in the CPT group were 63 years old. Similarly, both groups are unique concerning the body mass index, which falls between the ranges of 26 and 27. Thirty-five percent of patients belonging to the KT + CPT group had symptoms for more than 12 months. However, 45% of patients in the CPT group experienced symptoms for 3–6 months. Degenerative changes were mentioned as a major causative factor by most of the patients in both groups, as well as a history of pain during sleep. The mean and standard deviation of measures such as the pain scale, knee ROM, WOMAC pain, stiffness, difficulty, and total score, as well as the TUG test, are shown in Table 2 for both the KT plus CPT and CPT groups.

**Table 1:** Demographic measures are presented as mean and standard deviations for both treatment groups

**Demographic measures**	**Kinesio taping plus conventional PT group** Mean ± standard deviation	**Conventional PT group** Mean ± standard deviation
Age	60.60 ± 9.43	63.40 ± 7.98
Smoking	1.50 ± 0.51	1.45 ± 0.51
Causative factor	1.55 ± 1.14	1.35 ± 0.67
BMI	26.65 ± 2.85	27.72 ± 2.41
Dominant	1.70 ± 0.66	1.60 ± 0.60
Duration	2.00 ± 0.86	1.80 ± 0.83
Occurrence	1.75 ± 0.44	1.90 ± 0.31
Type of treatment	1.55 ± 1.15	1.60 ± 1.14
Sleep pain	1.45 ± 0.51	1.40 ± 0.50

*PT* physical therapy, *BMI* body mass index

**Table 2:** Mean and standard deviation of all the outcome parameters at 0 weeks, 3 weeks, and 6 weeks

**Outcome measures**		**Kinesio taping plus conventional PT group**			**Conventional PT group**		
		0 weeks Mean ± SD	3 weeks Mean ± SD	6 weeks Mean ± SD	0 weeks Mean ± SD	3 weeks Mean ± SD	6 weeks Mean ± SD
Pain scale		6.35 ± 1.23	4.20 ± 1.15	3.10 ± 1.12	6.80 ± 1.32	5.10 ± 1.17	4.05 ± 1.15
Knee ROM	Flexion	120.85 ± 8.20	128.55 ± 5.41	129.70 ± 5.13	122.70 ± 8.27	129.45 ± 6.93	131.85 ± 5.25
	Extension	0.45 ± 0.94	0.20 ± 0.62	0.00 ± 0.00	0.30 ± 0.73	0.25 ± 0.64	0.00 ± 0.00
WOMAC score	Pain	10.15 ± 2.41	5.15 ± 1.69	4.15 ± 1.39	9.85 ± 4.07	8.50 ± 3.52	7.35 ± 2.74
	Stiffness	4.30 ± 1.75	3.10 ± 1.48	2.00 ± 0.92	4.95 ± 1.36	3.30 ± 1.03	2.90 ± 1.02
	Difficulty	31.05 ± 8.35	16.40 ± 6.72	15.80 ± 6.63	23.85 ± 4.11	17.95 ± 6.39	16.95 ± 5.17
	Total	45.50 ± 11.36	21.9 ± 6.53	21.95 ± 7.67	37.95 ± 6.19	29.65 ± 8.82	27.6 ± 7.05
TUG test		9.55 ± 1.13	8.80 ± 1.47	8.08 ± 1.21	9.13 ± 1.40	8.65 ± 1.35	8.15 ± 1.20

*PT* Physical therapy, *SD* standard deviation, *ROM* range of motion, *WOMAC* Western Ontario and McMaster Universities Osteoarthritis Index, *TUG* Timed Up and Go test

Table 3 shows that all variables, including pain scale, knee ROM, WOMAC pain, stiffness, difficulty, and total score, and the TUG test in the KT + CPT group, showed a substantial difference in the pre, mid, and post-treatment scores (*p *< 0.05). Moreover, a significant difference was observed in the CPT group concerning the pain scale, knee flexion ROM, and WOMAC stiffness scores measured at three different intervals (*p *< 0.05). Following the identification of significant differences, a Tukey HSD post hoc test was used to compare the scores of all parameters in the two-time intervals to see whether a significant variation occurs within the group.

**Table 3:** Analysis of pain, knee ROM, WOMAC, and TUG tests of subjects in both experimental groups (ANOVA)

**Outcome variables**		**Kinesio taping plus conventional PT group**				**Conventional PT group**			
		Sum of squares	df	Mean square	*F* (*p*-value)	Sum of squares	df	Mean square	*F* (*p*-value)
Pain scale	Between weeks	109.300	2	54.650	40.168* (p < 0.000)	77.033	2	38.517	26.152* (p < 0.000)
	Within weeks	77.550	57	1.361		83.950	57	1.473	
Knee ROM-flexion	Between weeks	926.233	2	463.117	11.312* (p < 0.000)	900.300	2	450.150	9.375* (p < 0.000)
	Within weeks	2333.700	57	40.942		2735.700	57	47.995	
Knee ROM-extension	Between weeks	2.033	2	1.017	2.400^NS^ (p = 0.100)	1.033	2	0.517	1.641^NS^ (p = 0.203)
	Within weeks	24.150	57	0.424		17.950	57	0.315	
WOMAC score-pain	Between weeks	413.333	2	206.667	58.418* (p < 0.000)	19.633	2	9.817	0.990^NS^ (p = 0.378)
	Within weeks	201.650	57	3.538		565.100	57	9.914	
WOMAC score-stiffness	Between weeks	52.933	2	26.467	13.005* (p < 0.000)	47.233	2	23.617	17.961* (p < 0.000)
	Within weeks	116.000	57	2.035		74.950	57	1.315	
WOMAC score-difficulty	Between weeks	2983.633	2	1491.817	28.167* (p < 0.000)	356.133	2	238.067	3.879^NS^ (p = 0.073)
	Within weeks	3018.950	57	52.964		1204.450	57	26.148	
WOMAC score-total	Between weeks	7410.433	2	3705.217	48.243* (p < 0.000)	1001.433	2	600.717	4.136^NS^ (p = 0.092)
	Within weeks	4377.750	57	76.803		2950.300	57	55.268	
TUG test	Between weeks	21.612	2	10.806	6.571* (p < 0.003)	9.703	2	4.852	2.787^NS^ (p = 0.070)
	Within weeks	93.742	57	1.645		99.225	57	1.741	

*NS* nonsignificant

*p-value < 0.05 shows significant

The pain scale had a significant mean difference between the time intervals as shown in Table 4, namely the first and third weeks, the third and sixth weeks, and the first and sixth weeks in both groups. In the KT + CPT group, the mean difference observed between the first and third weeks and the first and sixth weeks was statistically significant in terms of knee flexion ROM, WOMAC pain, stiffness, difficulty, and total scores. In the KT + CPT group, the mean difference in TUG test values recorded between the first and sixth weeks similarly showed a significant value at the 0.05 level. Between the first and third weeks, the third and sixth weeks, and the first and sixth weeks in either group, there was no significant difference in the mean scores of knee extension ROM. Furthermore, the mean difference between the first and third weeks, as well as the first and sixth weeks, in the CPT group, was statistically significant at the 0.05 level in terms of knee flexion ROM, WOMAC stiffness, difficulty, and overall scores. Additionally, the mean difference measurement between the first and third weeks, third and sixth weeks, and first and sixth weeks did not show any significant improvement concerning the WOMAC pain score and TUG test in the CPT group (*p *< 0.05).

**Table 4:** Tukey HSD post hoc test of outcome parameters among different time intervals in both groups

Outcome variables	Mean		**Kinesio taping plus conventional PT group**		**Conventional PT group**	
			MD	Significant (*p*-value)	MD	Significant (*p*-value)
PAIN scale	Pre	Mid	2.15*	0.000	1.70*	0.000
	Pre	Post	3.25*	0.000	2.75*	0.000
	Mid	Post	1.10*	0.012	1.05*	0.022
Knee ROM-flexion	Pre	Mid	7.70*	0.001	6.75*	0.009
	Pre	Post	8.85*	0.000	9.15*	0.000
	Mid	Post	1.15	0.837	2.40	0.521
Knee ROM-extension	Pre	Mid	0.25	0.450	0.05	0.957
	Pre	Post	0.45	0.082	0.30	0.218
	Mid	Post	0.20	0.598	0.25	0.343
WOMAC score-pain	Pre	Mid	5.00*	0.000	0.75	0.733
	Pre	Post	6.00*	0.000	1.40	0.345
	Mid	Post	1.00	0.221	0.65	0.792
WOMAC score-stiffness	Pre	Mid	2.30*	0.000	1.65*	0.000
	Pre	Post	1.20*	0.002	2.05*	0.002
	Mid	Post	1.10*	0.004	0.40	0.516
WOMAC score-difficulty	Pre	Mid	14.65*	0.000	5.90*	0.002
	Pre	Post	15.25*	0.000	6.90*	0.000
	Mid	Post	0.60	0.967	1.00	0.823
WOMAC score-total	Pre	Mid	23.60*	0.000	8.30*	0.002
	Pre	Post	23.55*	0.000	10.35*	0.000
	Mid	Post	0.05	1.000	2.05	0.660
TUG test	Pre	Mid	0.75	0.163	0.48	0.480
	Pre	Post	1.47*	0.002	0.98	0.056
	Mid	Post	0.72	0.187	0.50	0.459

0 weeks, pre; 3 weeks, mid; and 6 weeks, post-measurement scores

*MD mean difference

*p*-value < 0.05 shows significant

## Discussion

Our research results showed the majority of knee OA patients tolerated the 6-week course of Kinesio Tex Gold FP (5 cm × 5 m) knee taping very well. There were no significant adverse effects or functional deterioration that would force the intervention to be discontinued. Clinically relevant improvements in pain, knee flexion ROM, and the functional activities of WOMAC and Timed Up and Go tests were seen in the KT with CPT group. However, although we found the CPT group alone showed significant improvement in pain reduction and knee flexion ROM, at the same time, this group did not produce any detectable benefit in knee functioning as assessed by WOMAC and TUG tests.

Within the scope of these principles, two different therapy approaches (KT plus CPT and CPT alone) were chosen for management in this study. Baseline demographic and clinical outcome variables demonstrate a homogenous presentation among the study groups (*p* > 0.05). The chosen KT method is suitable for our treatment application based on the clinical findings. Our study results showed that KT plus CPT can reduce pain in knee OA patients. The same study report was revealed to the authors of their study that, in a geriatric population, the KT with CPT group showed more significant relief in knee pain after 3 weeks of intervention than the CPT group [29]. The lifting effect of KT also creates more space between the dermis layer and the muscle. By lowering pressure on pain receptors beneath the skin, the additional space is claimed to reduce pain [7]. These results show that, when compared to other therapies, KT can help individuals with knee OA relieve pain and improve joint function. Even though most included trials found KT to be useful in the management of knee OA, there were a few studies that did not show it to be useful for knee OA [30]. Thus, the author claimed that three consecutive days of KT application resulted in pain relief, joint stiffness reduction, and enhanced knee function [31], but this was contradicted by a trial conducted by Rahlf et al. in 2018 [32]. As a result, more investigation into the true underlying mechanism for the beneficial taping benefits is needed. Aside from the KT application, the researchers intend to add transcutaneous electrical nerve stimulation (TENS) as a combination therapy to see benefits from the treatment. The function of the TENS increases adrenocorticotropic hormone production and plasma levels, as well as beta-endorphin levels. Previous research has shown that TENS can reduce the need for analgesics for postoperative pain [33]. In our study, KT with TENS, statistically significant improvements were observed in the VAS pain scale in patients with KT and CPT at 6 weeks of treatment.

Patients with knee OA may have joint stiffness, which can lead to a reduction in range of motion. The evidence for the positive effects of exercise on knee OA was determined to be of high to moderate quality in a Cochrane comprehensive review [10]. Besides the above, KT may provide tactile stimulation and mechanical support as long as the patients insist on doing their exercises. Exercise is well known for reducing pain, strengthening muscles, and improving balance control around the affected knee joint. In addition, it reduces joint space narrowing, increases the proteoglycan content in the cartilage, and also has disease-modifying benefits [34]. Both groups improved their knee flexion ROM statistically significantly in our study, and the beneficial effect lasted for six weeks, with a lesser amount of extension deficiency. A similar study found that knee flexion improved, but knee extension remained unchanged [8]. In contrast to our findings, the authors found no evidence of a favorable effect of KT on ROM. However, KT, on the other hand, is commonly used to relieve pain, edema, and inflammation, provide mechanical support, increase range of motion, and improve gait patterns, and patient functional outcomes [8].

As knee OA progresses, a vicious circle of pain–weakness–pain develops, resulting in functional deficits, decreased ROM, and a loss of muscle strength [8]. When compared to the placebo group, previous meta-analyses concluded that the KT group showed significant improvements in self-reported pain during activity, knee-related health status, and proprioceptive sensibility [35]. Certainly, the knee WOMAC scores are routinely used in assessing knee OA patients’ activities of daily living. In our study, the WOMAC subscales and total scores in the KT plus CPT group who received the treatments weekly twice showed significant improvement when compared to the WOMAC scores in the CPT group. However, only at 0–3 and 0–6 weeks did the KT with CPT group show a substantial improvement in WOMAC pain, stiffness, and total scores. The results of follow-up investigations with 3,705 patients with knee difficulties revealed that the pain indices were significantly reduced among the WOMAC subitems, according to the other study [36]. Based on the findings, KT application stimulates the muscles surrounding the knee joint, where abnormal muscle tonus develops as a result of wear and tear and articular cartilage degeneration. This aids muscle homeostasis and gradually reduces pain and stiffness, preventing the muscle tonus state from worsening and allowing knee joint function to improve [37].

The TUG test is recommended by the International Osteoarthritis Research Society as a performance-based measure of physical function in subjects with hip and knee OA [25]. Furthermore, among sit-to-stand tests for hip/knee OA, the TUG test was shown to have the best evidence of measurement [25]. Our study shows a remarkable improvement in KT plus CPT groups in terms of TUG durations of 0–6 weeks. The KT plus exercise group improved more than the exercise group alone in terms of ambulatory performance in knee OA, according to the study by Castrogiovanni et al. in 2016 [38]. As a result, the findings of the prior study are compatible with our findings. In conclusion, KT combined with CPT is more effective than conventional physical therapy alone in treating knee OA in terms of pain reduction, increase range of motion, and improved physical function activities.

## Recommendations

Large sampling, well-structured study design, and randomized controlled trials in multiple centers are needed to assess the long-term effects of Kinesio taping combined with conventional physical therapy versus conventional physical therapy alone for knee OA.

## Study limitations

The study sample was limited to the male gender only. There was also no control group, so the improvement in the usual care group could be attributed to normal resolution.

## Conclusions

Kinesio taping plus conventional physical therapy showed statistically significant effects on pain reduction, increased range of motion, and improved functional activity in the third week and was similarly effective in the sixth week of treatment in patients with knee osteoarthritis. TENS and exercise programs for knee osteoarthritis patients have also been proven to be safe and effective, mainly reducing pain, and improving ROM. Kinesio taping could be an alternate therapy option for knee OA, particularly if an immediate effect is desired.
